# The Distribution Pattern of Ocular Residual Astigmatism in Chinese Myopic Patients

**DOI:** 10.3389/fmed.2022.763833

**Published:** 2022-05-24

**Authors:** Changting Tang, Zijing Du, Guanrong Wu, Xuanyun Tan, Siwen Zang, Honghua Yu, Yijun Hu

**Affiliations:** ^1^Guangdong Eye Institute, Department of Ophthalmology, Guangdong Provincial People's Hospital, Guangdong Academy of Medical Sciences, Guangzhou, China; ^2^The Second School of Clinical Medicine, Southern Medical University, Guangzhou, China; ^3^Affiliated Hospital of Guilin Medical University, Guilin, China; ^4^Aier Institute of Refractive Surgery, Refractive Surgery Center, Guangzhou Aier Eye Hospital, Guangzhou, China; ^5^Aier School of Ophthalmology, Central South University, Changsha, China

**Keywords:** ocular residual astigmatism, manifest astigmatism, myopia, refractive surgery, astigmatism correction

## Abstract

**Purpose:**

We aimed to investigate the distribution of ocular residual astigmatism (ORA) and its associations with age, gender, manifest refraction, and other ocular indicators in Chinese patients with myopia.

**Design:**

This is a multi-center retrospective cross-sectional study.

**Method:**

The study included 7,893 patients with myopia (7,893 eyes) aged 18–40 years from five ophthalmic centers. Anterior segment biometrics of the eyes were collected from the Pentacam. ORA and its summated vector mean were calculated using Alpins vector analysis. Compensation factor (CF) was used to evaluate the relation between ORA and corneal astigmatism. ORA in different age, gender, and refraction groups was compared. The Spearman correlation was adopted to reveal multiple ocular indicators associated with ORA, which were integrated into a multiple linear regression model to predict ORA.

**Results:**

Distribution of ORA was slightly positively skewed (Skewness= 2.111, Kurtosis = 19.660, KS *P* < 0.0001). Mean ORA was 0.74 ± 0.39 D (95% normal range: 0.14–1.54 D). Among all the subjects, 22.4% of the eyes had an ORA magnitude of 1.0 D or more. Undercompensation or full-compensation of anterior corneal astigmatism (ACA) by ORA prevailed in both J_0_ (76.99%) and J_45_ (58.48%). Women had higher ORA power than men (0.77 ± 0.36 D vs. 0.73 ± 0.41 D, *P* < 0.0001). Participants with less negative spherical equivalent (SE) or higher manifest astigmatism (MA) also had higher ORA (all *P* < 0.0001). ORA was significantly correlated with ACA (*r* = 0.405) and posterior corneal astigmatism (PCA, *r* = 0.356). The multivariate logistic regression analysis showed strong predictability of ORA magnitude >1.0 D using anterior segment parameters (area under the receiver operating characteristic curve: 0.739).

**Conclusion:**

ORA is present in Chinese adults with myopia and is affected by multiple ocular factors. Our findings may provide valuable information about ORA distribution in candidates for refractive surgery, helping optimize the outcome of astigmatism correction.

## Introduction

It is well-known that the ocular manifest astigmatism (MA) is comprised of the anterior corneal astigmatism (ACA) and the ocular residual astigmatism (ORA). ORA mainly stems from the posterior cornea, aqueous humor, crystalline lens, and some perceptual retinal components (1, 2). Usually, we use corneal topography to calculate ACA based on simulated corneal curvature and obtain MA by retinoscopy or phoropter. Clinically, the magnitude and orientation of MA may not always match with those of ACA. ORA is known as the vectoral difference between ACA and MA at the corneal plane (3, 4). This difference may result in excess corneal astigmatism and resultant glare, starburst, and haloes (GASH), leading to reduced visual acuity and even visual fatigue after refractive surgery (5–7). Therefore, appropriate management of ORA has become increasingly important.

In corneal refractive surgery, astigmatism correction is often performed based on the preoperative MA only or ACA only (8). These approaches may lead to suboptimum postoperative visual quality if patients have a significant ORA (9, 10). A large number of studies have suggested that astigmatism correction by laser *in situ* keratomileusis (LASIK) can achieve a better correction outcome if the preoperative MA mainly comes from ACA (4, 5, 11). Results in the previous studies also indicated that the efficacy of LASIK surgery in eyes with low ORA was more than two times as good as in eyes with high ORA (5, 12). Thus, estimation of the ORA in patients with myopia having refractive surgery may be helpful for achieving a satisfactory postoperative visual acuity. In addition, ORA also has a significant influence on cataract surgery. Traditionally, astigmatism correction in cataract surgery mainly relies on the amount of total corneal astigmatism, which is usually calculated based on ACA (13). A growing number of studies have reported that patients may suffer from relatively low-visual quality after implantation of intraocular lenses (IOLs) if the patients have a substantial posterior corneal astigmatism (PCA), which is one of the components of ORA (14–16). The Barrett toric algorithm based on the measured PCA shows better predictability than those that do not consider the contribution of PCA in IOL power calculation (17, 18). Whether performing corneal refractive surgery or cataract surgery, taking ORA into astigmatism correction is of significant importance. Therefore, it would be valuable to reveal the details about the distribution of ORA before surgery for precise surgical management of astigmatism.

Although it is well-known that ORA is a vital factor affecting postoperative visual quality, so far there have been few research on the ORA distribution pattern of myopic adults in China, a country with the largest refractive surgery population in the world. In this study, we collected data from five ophthalmic centers to investigate the distribution pattern of ORA and its associations with age, gender, manifest refraction, and other ocular indicators in Chinese adults with myopia. Our results could be of clinical significance and provide implications on astigmatism correction in myopic refractive surgery and cataract surgery.

## Methods

### Participants

This retrospective cross-sectional study was adherent to the tenets of the Declaration of Helsinki and approved by the Institutional Review Board (IRB) of Guangzhou Aier Eye Hospital (GZ), Shenyang Aier Eye Hospital (SY), Wuhan Aier Eye Hospital (WH), Chengdu Aier Eye Hospital (CD), and Hankou Aier Eye Hospital (HK). The IRBs decided to waive the necessity to get informed consent since our study was only a review of medical records from which patients could not be identified (19, 20). Patients with myopia who had refractive surgery (corneal laser surgery or ICL implantation) in the five ophthalmic centers between 2017 and 2020 and met the inclusion criteria were selected consecutively. Only the right eye of each patient was included for the analysis. The inclusion criteria were myopic eyes with spherical equivalent (SE) ≤ −0.50 D and good quality of Pentacam examination images. The exclusion criteria were coexisting corneal diseases, such as keratoconus, forme fruste keratoconus, previous ocular surgery or trauma, and uveitis, glaucoma, wearing soft contact lenses within 2 weeks or rigid gas-permeable lenses within 1 month before examination, and younger than 18 years (unstable refraction) or older than 40 years (significant change of ORA induced by crystalline lens) (19, 20).

### Examinations

All patients underwent detailed preoperative examinations, such as best-corrected visual acuity (BCVA), intraocular pressure (IOP), manifest and cycloplegic refraction (sphere and cylinder), slit-lamp examination of the anterior segment, corneal topography, and Pentacam measurements. We divided the eyes into four myopia groups according to the SE: low myopia (LM, −3.00 D < SE ≤ −0.50 D), moderate myopia (MM, −6.00 D < SE ≤ −3.00 D), high myopia (HM, −10.00 D < SE ≤ −6.00 D), and ultra-high myopia (UHM, SE ≤ −10.00 D), and four astigmatism groups according to the MA: slight astigmatism (SMA, MA < 0.50 D), low astigmatism (LMA, 0.50 D ≤ MA < 1.00 D), moderate astigmatism (MMA, 1.00 D ≤ MA < 2.00 D), and high astigmatism (HMA, MA ≥ 2.00 D).

Pentacam was used for all subjects by skilled technicians. The Pentacam instrument (Pentacam HR, Oculus GmbH, Wetzlar, Germany) was regularly calibrated every we-ek. We have described details and quality control of Pentacam examination in previous studies (19, 20). Anterior segment data were exported from the Pentacam machine. ACA and PCA were calculated using corneal curvature radii in the central 3-mm ring mode.

### Data Analysis and Calculation

In this study, ORA was calculated by subtracting ACA from MA using the “ASSORT vector calculator” (https://assort.com/assort-vector-calculator) based on Alpins vector analysis, and the summated vector mean was obtained by adding each vector then dividing the resultant vector's magnitude by the number of vectors (21–23). All the astigmatism data were converted into power vector components using the power vector method (24). MA and ACA [cylinder (C), axis (θ)] were transformed into two dioptric components, including J_0_ (power of Jackson cross cylinder at 90° and 180°) and J_45_ (power of Jackson cross cylinder at 45 and 135 degrees) (25). J_0_ and J_45_ could be obtained from the following formulae, where θ was the cylindrical axis (25, 26):


(1) $$\begin{array}{r} J_{0}\;=\;\left(-\frac{C}{2}\;\right)\cos\;\left(2\theta \right) \end{array}$$

(2) $$\begin{array}{r} J_{45}\;=\;\left(-\frac{C}{2}\;\right)\sin\left(2\theta \right) \end{array}$$

In this study, the compensation factor (CF) was used to evaluate the compensation effect of ORA on ACA in J_0_ and J_45_. CF was the ratio of ORA and ACA calculated by the methods as described by Muftuoglu et al. (27): $CF=\frac{\left(corneal\;astigmatism\;-\;refractive\;astigmatism\right)}{corneal\;astigmatism}.\;$The compensation effect was classified into six types (25, 27): same axis augmentation (CF < −0.1), no compensation (CF from −0.1 to 0.1), undercompensation (CF from 0.1 to 0.9), full compensation (CF from 0.9 to 1.1), overcompensation (CF from 1.1 to 2), and opposite axis augmentation (CF more than 2). Undercompensation indicates that MA is smaller than ACA in magnitude while having the same axis as ACA. Overcompensation means that MA magnitude is smaller than that of ACA but the axis is at the opposite angle of ACA. Same axis augmentation indicates that the magnitude of MA is larger than that of ACA while having the same axis as ACA. Opposite axis augmentation means the magnitude of MA is larger than that of ACA but the axis is at the opposite angle of ACA (25, 28).

### Statistical Analysis

All data analyses were performed using SPSS version 22.0 software (IBM Corporation, Armonk, NY, USA). The normality of all variables was evaluated by the Kolmogorov–Smirnov (KS) test, and the Kruskal–Wallis test was used to compare the variables among different groups. Correlations between anterior segment parameters and ORA were evaluated by the Spearman correlation test, and variables significantly correlated with ORA were tested by univariable logistic regression analysis for the association between these variables and high ORA (>1.0 D). Variables significantly associated with high ORA in the univariable logistic regression and not having colinearity with other variables were included in a multivariate logistic regression model to analyze the association between these variables and high ORA. The diagnostic ability of the multivariate logistic regression model was determined using the receiver operating characteristic (ROC) curve analysis. Statistically significant differences were set as *p*-value <0.05.

## Result

This study included 7,893 eyes of 7,893 patients with myopia from five ophthalmic centers (2,340 patients from GZ, 2,255 patients from SY, 1,480 patients from CD, 1,511 patients from WH, and 307 patients from HK). There were 4,416 men (55.9%) and 3,477 women (44.1%) with a mean age of 25.14 ± 5.41 years. The mean SE and mean MA of the eyes were −5.13 ± 2.05 D and 0.69 ± 0.62 D, respectively. Age, gender, SE, MA, ACA, and ORA in the five ophthalmic centers were significantly different (all *P* < 0.0001). Demographic data of the eyes are listed in Table 1.

**Table 1:** Demographics of the patients in the five ophthalmic centers.

**Demographics**	**Ophthalmic centers**
	**GZ**	**SY**	**WH**	**CD**	**HK**	**Pooled**	***P*** -Value^*^
Patients^a^	2,340 (29.6%)	2,255 (28.6%)	1,511 (19.1%)	1,480 (18.8%)	307 (3.9%)	7,893 (100.0%)	N/A
Male^a^	1,086 (24.6%)	1,462 (33.1%)	762 (17.3%)	910 (20.6%)	196 (4.4%)	4,416 (55.9%)	<0.001
Female^a^	1,254 (36.1%)	793 (22.8%)	749 (21.5%)	570 (16.4%)	111 (3.2%)	3,477 (44.1%)	<0.001
Age (years)^b^	26.94 ± 5.42	23.88 ± 5.15	25.39 ± 5.03	24.19 ± 5.46	23.97 ± 4.78	25.14 ± 5.41	<0.001
SE (D)^b^	−5.17 ± 2.18	−4.81 ± 1.71	−5.28 ± 1.93	−5.27 ± 2.23	−5.65 ± 2.68	−5.13 ± 2.05	<0.001
MA (D)^b^	0.74 ± 0.67	0.70 ± 0.62	0.63 ± 0.55	0.69 ± 0.64	0.71 ± 0.58	0.69 ± 0.62	<0.001
95% normal range MA (D)	0–2.50	0–2.25	0–2.00	0–2.25	0–2.00	0–2.25	<0.001
ACA (D)^b^	1.22 ± 0.70	1.29 ± 0.66	1.09 ± 0.63	1.21 ± 0.70	1.23 ± 0.73	1.21 ± 0.68	<0.001
95% normal range ACA (D)	0.19–2.86	0.23–2.74	0.13–2.55	0.18–2.90	0.20–2.97	0.19–2.79	<0.001
ORA (D)^b^	0.75 ± 0.40	0.73 ± 0.37	0.77 ± 0.43	0.70 ± 0.36	0.83 ± 0.44	0.74 ± 0.39	<0.001
95% normal range ORA (D)	0.14–1.58	0.15–1.47	0.15–1.65	0.12–1.45	0.15–1.90	0.14–1.54	<0.001

a *Presented as number (%)*.

b *Presented as mean ± standard deviation*.

* *Comparison among the five ophthalmic centers using the Kruskal-Wallis test*.

*SE, spherical equivalent; D, diopter; MA, manifest astigmatism; ACA, anterior corneal astigmatism; ORA, ocular residual astigmatism; GZ, Guangzhou Aier Eye Hospital; SY, Shenyang Aier Eye Hospital; CD, Chengdu Aier Eye Hospital; WH, Wuhan Aier Eye Hospital; HK, Hankou Aier Eye Hospital*.

**Figure 1:**
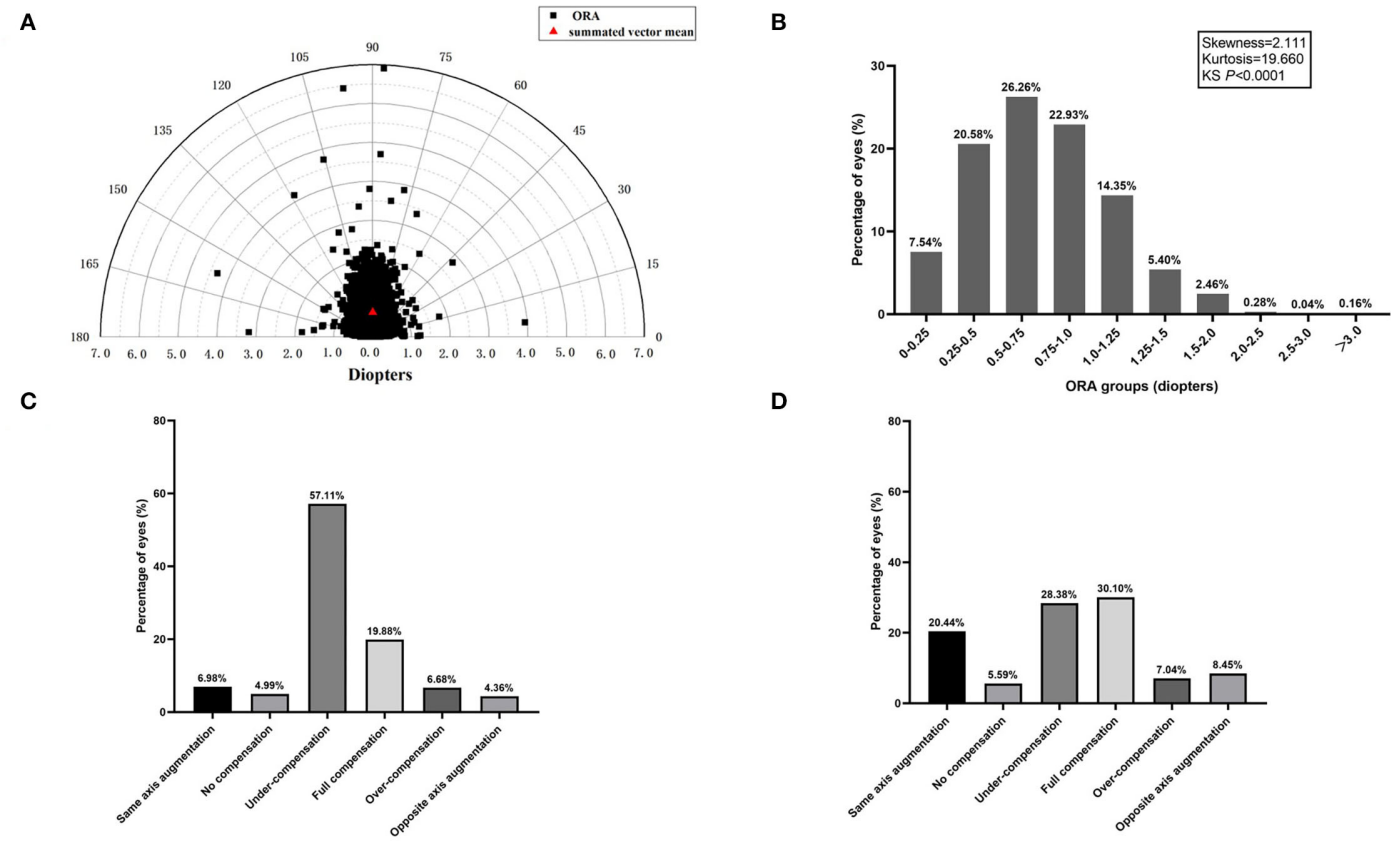
**(A)** The distribution of ocular residual astigmatism (ORA) magnitude and orientation is shown as a polar diagram. **(B)** Frequency distribution of ORA magnitude. **(C)** Percentages of anterior corneal astigmatism (ACA) compensation by ORA in J_0_. **(D)** Percentages of ACA compensation by ORA in J_45_.

The magnitude and orientation of ORA in all of the eyes were plotted as a polar diagram (Figure 1A). Distribution of ORA was slightly positively skewed (Skewness = 2.111, Kurtosis = 19.660, KS *P* < 0.0001). Mean ORA was 0.74 ± 0.39 D (95% CI: 0.74–0.75 D), and summated vector mean was 0.63 D Ax 89, and 22.4% of the eyes had an ORA magnitude of 1.0 D or more (Figure 1B).

Details about the compensation effect of ORA on ACA in J_0_ and J_45_ were shown in Figures 1C,D. There were five compensation types. Undercompensation or full compensation of ACA by ORA prevailed in both J_0_ (76.99%) and J_45_ (58.48%). As illustrated, in 76.99% of the eyes, J_0(ACA)_ were partially or fully compensated by J_0(ORA)_ and the percentage for J_45_ was 58.48%. Same axis augmentation and opposite axis augmentation in J_0_ were observed in 6.98 and 4.36% of eyes, respectively, and the percentage for J_45_ was 20.44 and 8.45%, respectively. Interestingly, no compensation in J_0_ was observed in 4.99% of eyes and the percentage for J_45_ was 5.59%.

**Figure 2:**
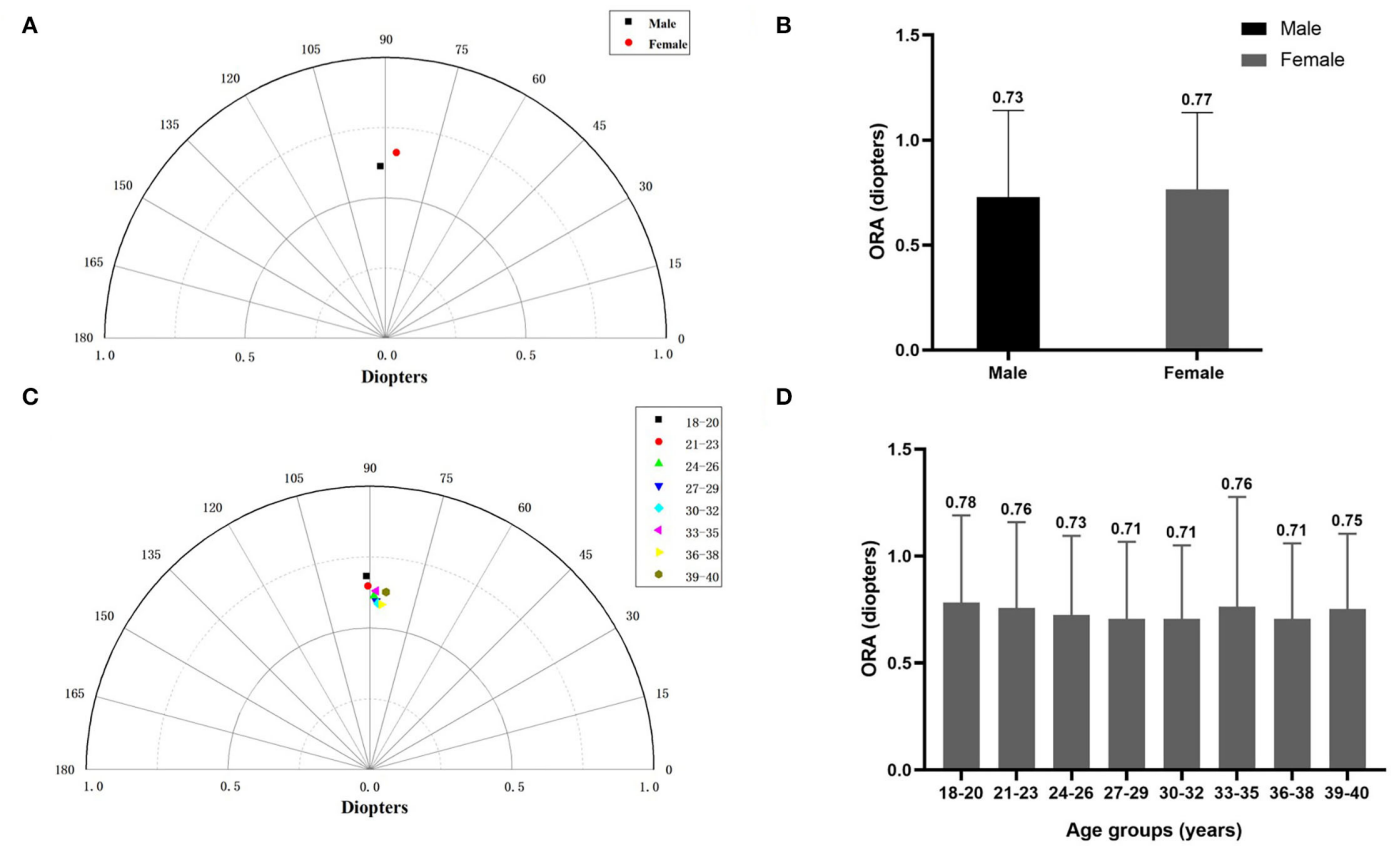
**(A)** The summated vector mean of ocular residual astigmatism (ORA) in different gender groups. **(B)** The mean magnitude of ORA in different gender groups. **(C)** The summated vector mean of ORA in different age groups. **(D)** The mean magnitude of ORA in different age groups.

Women had slightly higher ORA than men (summated vector mean: 0.66 D Ax 87 vs. 0.61 D Ax 92 and mean ORA: 0.77 ± 0.36 D vs. 0.73 ± 0.41 D, *P* < 0.0001) (Figures 2A,B). The change of ORA with aging was inconsistent (Figures 2C,D).

**Figure 3:**
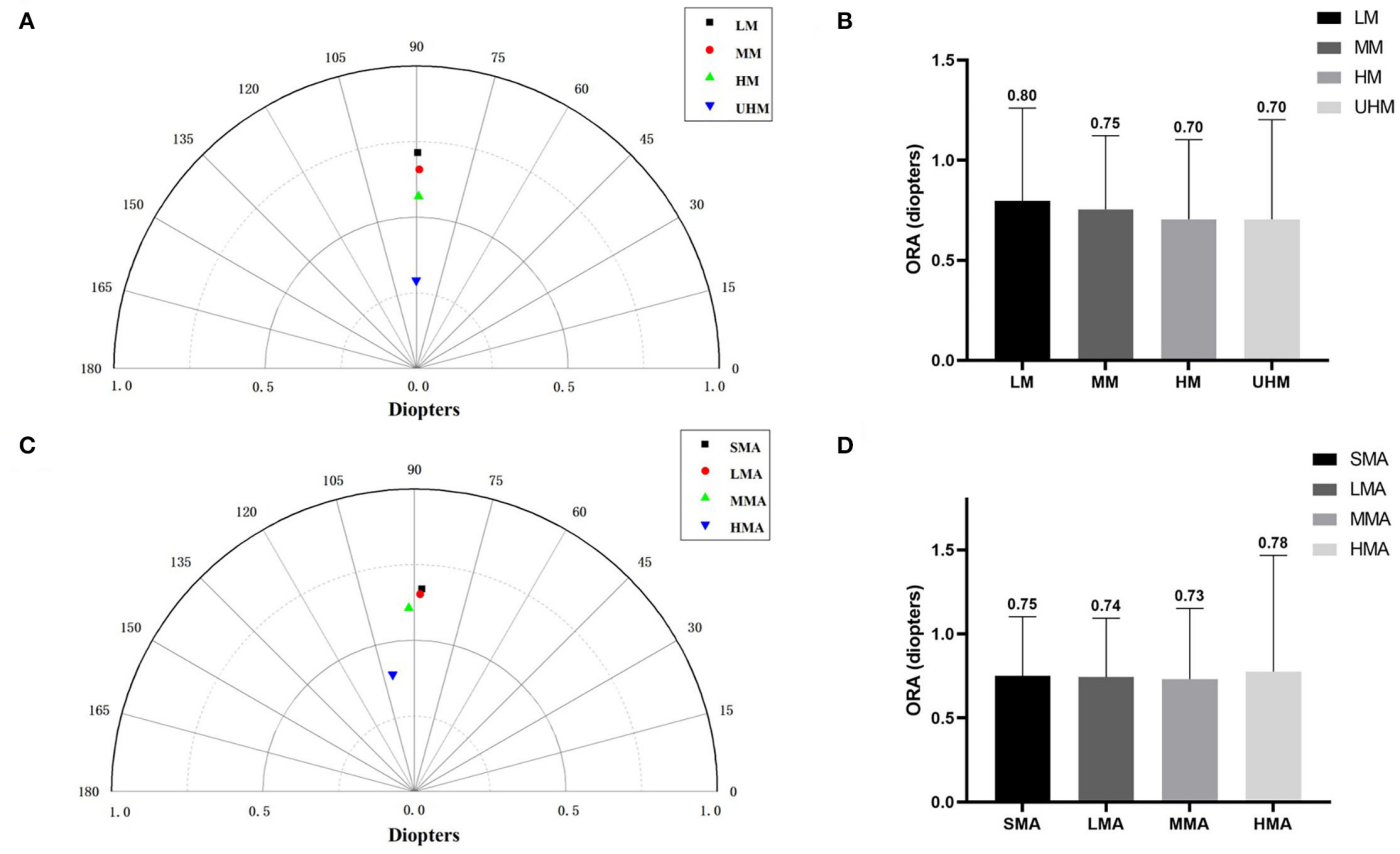
**(A)** The summated vector mean of ocular residual astigmatism (ORA) in different myopia groups. **(B)** The mean magnitude of ORA in different myopia groups. **(C)** The summated vector mean of ORA in different astigmatism groups. **(D)** The mean magnitude of ORA in different astigmatism groups.

In four myopia subgroups, the number of eyes with LM, MM, HM, and UHM were 945, 4,524, 2,272, and 152, respectively. In four astigmatism subgroups, the number of eyes with SMA, LMA, MMA, and HMA were 2,612, 2,958, 1,928, and 395, respectively. With an increasing degree of myopia and refractive astigmatism, the summated vector mean showed a downward trend (Figures 3A,C). The mean ORA was 0.80 ± 0.46 D in eyes with LM, 0.75 ± 0.37 D in eyes with MM, 0.70 ± 0.40 D in eyes with HM, and 0.70 ± 0.50 D in eyes with UHM (Figure 3B). It was interesting that eyes with an SE ≤ −6.00 D had lower ORA compared with those with an SE > −6.00 D, while eyes with an MA ≥ 2.00 D had higher ORA compared with those with an MA < 2.00 D (all *P* < 0.0001) (Figure 3D).

Correlation coefficients between ORA and other corneal biometrics are shown in Table 2. Among all the enrolled eyes, ORA was positively correlated with ACA (*r* = 0.405, *P* < 0.001) and PCA (*r* = 0.356, *P* < 0.001).

**Table 2:** Summary of Spearman's correlation analyses between ORA with other anterior segment parameters.

**Parameters**	**ORA**		
	***r***	**95% CI**	***P*-value**
KmF	0.171	(0.149, 0.192)	<0.001
KmB	−0.213	(−0.235, −0.192)	<0.001
Anterior corneal astigmatism	**0.405**	**(0.386, 0.425)**	**<0.001**
Anterior corneal eccentricity	0.149	(0.127, 0.170)	<0.001
Anterior corneal asphericity	−0.157	(−0.178, −0.136)	<0.001
Posterior corneal astigmatism	**0.356**	**(0.336, 0.376)**	**<0.001**
Posterior corneal eccentricity	0.068	(0.046, 0.090)	<0.001
Posterior corneal asphericity	−0.108	(−0.129, −0.086)	<0.001
Pachy apex	0.071	(0.049, 0.093)	<0.001
Corneal volume −3 mm	0.077	(0.055, 0.099)	<0.001
Corneal volume −5 mm	0.097	(0.075, 0.119)	<0.001
Corneal volume −7 mm	0.115	(0.093, 0.137)	<0.001
Corneal diameter	−0.02	(−0.025, 0.20)	0.838
Anterior chamber depth	−0.080	(−0.102, −0.058)	<0.001
Anterior chamber volume	−0.139	(−0.161, −0.117)	<0.001

*ORA, ocular residual astigmatism; KmF, mean anterior corneal curvature; KmB, mean posterior corneal curvature; CI, confidence interval. The bold values represent the correlation coefficient > 0.3*.

**Figure 4:**
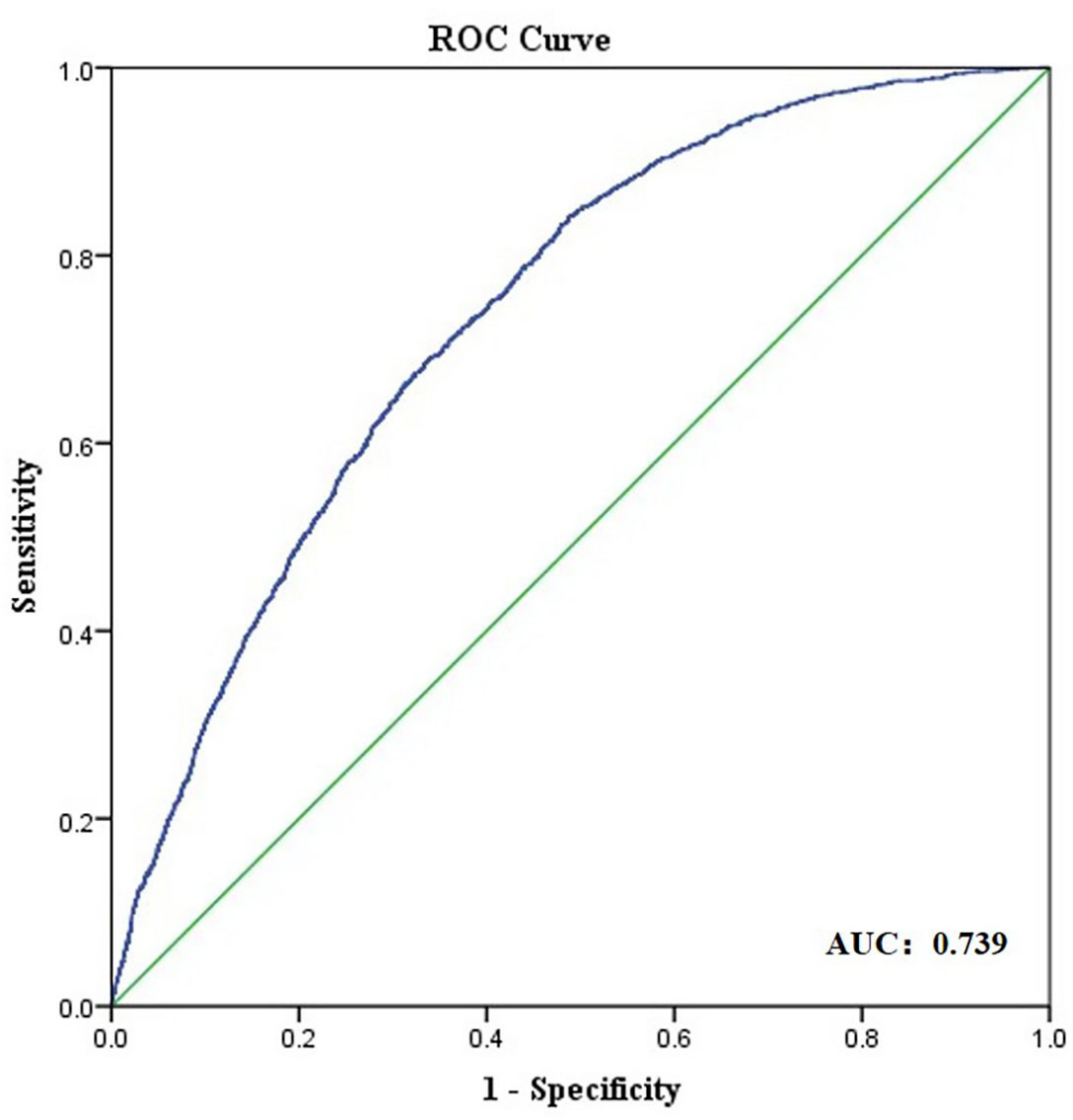
Receiver operating characteristic (ROC) curve obtained for anterior segment parameters as predictors of ocular residual astigmatism (ORA) larger than 1.0 D.

Results of the univariate and multivariate logistic regression analysis are shown in Table 3. In the multivariate logistic regression model, higher ACA and higher PCA were associated with high ORA [odds ratio (OR): 2.607, 95% CI:1.836–2.327 and OR: 6.921, 95% CI:3.812–12.566, respectively]. The area under ROC curve was 0.739 for the prediction of ORA > 1.0 D (Figure 4).

**Table 3:** Logistic regression analyzing associated factors on ORA larger than 1.0 diopters.

**Variables**	**Univariable regression model**		**Multivariate regression model**	
	**OR (95% CI)**	***P*-value**	**OR (95% CI)**	***P*-value**
KmF	1.272 (1.226, 1.319)	<0.001	1.164 (1.115, 1.215)	<0.001
KmB	0.151(0.119, 0.193)	<0.001	–	–
Anterior corneal astigmatism	2.916 (2.686, 3.165)	<0.001	2.067 (1.836, 2.327)	<0.001
Anterior corneal eccentricity	8.555 (5.436, 13.464)	<0.001	–	–
Anterior corneal asphericity	0.103 (0.067, 0.159)	<0.001	0.569 (0.335, 0.966)	0.037
Posterior corneal astigmatism	147.617 (96.922, 224.829)	<0.001	6.921 (3.812, 12.566)	<0.001
Posterior corneal eccentricity	1.846 (1.320, 2.580)	<0.001	–	–
Posterior corneal asphericity	0.284 (0.193, 0.418)	<0.001	0.681 (0.421, 1.102)	0.118
Pachy apex	1.004 (1.003, 1.006)	<0.001	1.003 (1.001, 1.005)	0.002
Corneal volume −3 mm	1.965 (1.525, 2.530)	<0.001	–	–
Corneal volume −5 mm	1.343 (1.230, 1.467)	<0.001	–	–
Corneal volume −7 mm	1.184 (1.136, 1.234)	<0.001	–	–
Anterior chamber depth	0.616 (0.502, 0.757)	<0.001	1.296 (0.846, 1.985)	0.233
Anterior chamber volume	0.992 (0.991, 0.994)	<0.001	0.993 (0.990, 0.997)	<0.001

*OR, odds ratio; ORA, ocular residual astigmatism; KmF, mean anterior corneal curvature; KmB, mean posterior corneal curvature; CI, confidence interval*.

## Discussion

Precise astigmatism correction is essential for surgeons to achieve satisfactory postoperative outcomes. The ORA is one of the most important factors leading to suboptimum visual quality after refractive surgery or cataract surgery (3, 12, 14). Therefore, evaluation of patients' preoperative ORA and its affecting factors may have an impact on the accuracy of astigmatism correction.

To our knowledge, this multiple center study is the largest investigation of the influence of demographic factors and ocular parameters on ORA in patients having refractive surgery. We showed a mean ORA magnitude of 0.74 ± 0.39 D in our population. Our result was consistent with a previous study (11) comprising 2,991 eyes of patients with myopia scheduled for LASIK and revealing that the mean ORA was 0.75 ± 0.39 D. In the two studies, both conducted by Alpins which consisted of 100 patients with myopia having corneal refractive surgery (4, 29), and the preoperative mean ORA as determined by vector analysis was 0.81 ± 0.05 D and 0.73 ± 0.43 D, respectively. These little differences can be attributed to differences in the refraction method (whether cycloplegia or not) and different devices for the measurement of corneal astigmatism and also the differences in the ethnic characteristics and refractive status of the subjects. Among all subjects, 22.4% of the eyes had an ORA magnitude of 1.0 D or more, which was consistent with previous studies (4, 28) reporting an ORA magnitude larger than 1.0 D in about one-third of the eyes. A growing number of evidence have shown that postoperative patients with uncorrected astigmatism error of 1.0 D or even less had symptoms of blurred vision and eye fatigue (6, 7, 30). Thus, better management of ORA is important for surgeons to achieve satisfactory postoperative outcomes.

Previous research have revealed that the magnitude of the ACA is larger than that of the MA, indicating that the ORA partially compensates for the ACA, and these two balance each other to produce a better-quality retinal image (11). Hoffmann et al. (31) had shown that ORA was mainly against-the-rule astigmatism and had a negative impact on ACA, which was commonly with-the-rule astigmatism in eyes. Meanwhile, Lin (32) conducted a study enrolling 165 eyes and found that ORA in 84.8% of the eyes acted as an offset to ACA and in 15.2% of the eyes superimposed the ACA. Furthermore, different compensation types of ACA by ORA have been reported (25). In our study, we found that different types of compensation had effects of ORA on the ACA. As illustrated, in 76.99% of the eyes, J_0(ACA)_ was partially or fully compensated by J_0(ORA)_ and the percentage for J_45_ was 58.48%. These findings were similar to a previous study including 206 Chinese children with myopia and showing that partial or full compensation of ACA by ORA was observed in 83.50% of eyes for J_0_ and 58.25% of eyes for J_45_ (28). We also found that augmentation compensation was present in 11.34–28.89% of the eyes for J_0_ and J_45_, regardless of same axis or opposite axis augmentation. Our findings were analogous to the compensation pattern previously reported by Muftuoglu et al. (27) in which the augmentation was observed in 25% of eyes for J_0_ and 54% of eyes for J_45_ by comparing the corneal topography maps. Various mechanisms have been revealed to explain how ORA compensates ACA. As demonstrated before, ACA could be compensated by PCA to a variety of 25–30% (33). Other contributors may be associated with internal ocular factors including crystalline lens shape- and position-related mechanisms, and inherent geometry-driven mechanisms (34). All these findings suggested that ORA affected MA in most of the studied eyes. Thus, consideration of the compensation effect of ORA may be necessary for astigmatism correction in refractive surgery or cataract surgery.

For patients with high-preoperative ORA, vector analysis of both refractive and corneal topographic parameters is a proven method for optimizing the treatment of astigmatism. Alpins has suggested that ORA as the vector difference between ACA and MA should not be ignored in astigmatism correction, and using a targeting vector enables the incorporation of MA and ACA values into the treatment plan (4). When a large ORA is present preoperatively, Alpins and Stamatelatos demonstrated that leaving 60% of ORA on the cornea (instead of the customary 100%) and 40% in the wavefront refraction second-order component (instead of the customary 0%) resulted in a better astigmatism correction and visual outcome than the conventional treatment (8, 35). Individual vectorial analysis for planning astigmatism correction may enable surgeons to achieve a full astigmatism correction in patients with high ORA (21, 29).

In this study, women had slightly higher ORA power than men (0.77 ± 0.36 D vs. 0.73 ± 0.41 D), which was similar to previous studies (11, 28). However, there existed contradictory reports of the correlation of gender with ORA (25, 36) and there was no agreement on this point. Our study did not detect a consistent tendency of change in ORA magnitude with aging, while Piero et al. (37) reported a similar insignificant correlation between ORA and age (*r* = 0.11, *P* = 0.10). Considering the narrow age gap in our study, the alteration of ORA magnitude and orientation with aging in other age groups needs to be further investigated.

We found that eyes with low or moderate myopia had a higher ORA than eyes with high or ultra-high myopia. Consistently, a previous study showed a negative correlation between axial length and ORA magnitude (28). We also detected a statistically significant correlation between ORA magnitude and some anterior segment parameters, such as ACA and PCA, suggesting that eyes with a larger ACA might also have a higher ORA. Similar results were also reported by Muftuoglu et al. (27). Hence, in patients with higher ACA, taking ORA into consideration is of crucial importance for precise astigmatism correction.

Crystalline lens is known as “internal optics” and contributes to the total ORA. Hence, physiological or pathological changes in the crystalline lens may also have a significant effect on ORA. For instance, age-related cataracts, particularly cortical opacities, can cause significant changes in ORA, considering asymmetric changes in the refractive index within different parts of the crystalline lens (38, 39). Another scenario where ORA may be affected is the malposition of the crystalline lens, such as lens subluxation and ectopia lentis (40). A recent study also showed that the ORA was greater in a population with shorter axial length and larger lens thickness, as internal astigmatism mostly arose from surfaces of the crystalline lens (41).

Some limitations of our study should be mentioned. First, we did not include other measuring devices to demonstrate the inter-device variation of ORA measurements. Furthermore, the conclusion of our study may not be applied to teenagers or older people, since participants of our study are patients with myopia aged 18–40 years old. In older patients with cataracts, the calculation of ORA may be inaccurate due to uncorrected MA measurement. In addition, the distribution and compensation effect of ORA may also be different in emmetropic or hyperopic eyes. Further studies to solve these issues are highly recommended. At last, this is a cross-sectional study. The impact of ORA on astigmatism and visual outcomes after surgery needs to be further investigated by prospective studies.

In conclusion, different levels of ORA were present in candidates of myopic refractive surgery. Undercompensation or full compensation of ACA by ORA was observed in the majority of eyes. Our results may help surgeons identify patients with significant preoperative ORA to optimize the outcome of astigmatism correction in the refractive surgery.

## Data Availability Statement

The raw data supporting the conclusions of this article will be made available on reasonable request to huyijun@gdph.org.cn.

## Ethics Statement

The studies involving human participants were reviewed and approved by the Institutional Review Board (IRB) of Guangzhou Aier Eye Hospital (GZ), Shenyang Aier Eye Hospital (SY), Wuhan Aier Eye Hospital (WH), Chengdu Aier Eye Hospital (CD), and Hankou Aier Eye Hospital (HK). Written informed consent for participation was not required for this study in accordance with the national legislation and the institutional requirements.

## Author Contributions

YH, HY, and CT: conception and design. CT, ZD, GW, SZ, and XT: data collection and analysis and results discussion and commented on the manuscript. CT: manuscript writing. YH and HY: data interpretation and final manuscript revision. All authors approved the submitted manuscript to be published.

## Funding

This work was supported by Grant 81870663 from the National Natural Science Foundation of China (HY), Grant KJ012019087 of the Outstanding Young Talent Trainee Program of Guangdong Provincial People's Hospital (HY), Grant KJ012019457 from the GDPH Scientific Research Funds for Leading Medical Talents and Distinguished Young Scholars in Guangdong Province (HY), Grant Y012018145 from the Talent Introduction Fund of Guangdong Provincial People's Hospital (HY), Grant A2021378 from the Medical Scientific Research Foundation of Guangdong Province, China (YH), Grant 2018SK50106 from the Technology Innovation Guidance Program of Hunan Province (YH), and Grant AM1909D2 and AR1909D2 from the Science Research Foundation of Aier Eye Hospital Group (YH).

## Conflict of Interest

The authors declare that the research was conducted in the absence of any commercial or financial relationships that could be construed as a potential conflict of interest.

## Publisher's Note

All claims expressed in this article are solely those of the authors and do not necessarily represent those of their affiliated organizations, or those of the publisher, the editors and the reviewers. Any product that may be evaluated in this article, or claim that may be made by its manufacturer, is not guaranteed or endorsed by the publisher.
